# Effects of living in the same region as one’s workplace on the total fertility rate of working women in Korea

**DOI:** 10.4178/epih.e2019043

**Published:** 2019-10-09

**Authors:** Yeon-Yong Kim, Hee-Jin Kang, Seongjun Ha, Jong Heon Park

**Affiliations:** 1 Department of Big Data, National Health Insurance Service, Wonju, Korea; 2 Department of Benefits Strategy, National Health Insurance Service, Wonju, Korea

**Keywords:** Birth rate, Working women, Workplace, Population policy, Korea

## Abstract

**OBJECTIVES:**

The lowest-low fertility status of Korea has continued for the past 17 years despite governmental efforts to encourage childbirth. As the number of working women has increased, their residence patterns have changed; however, the impact of this factor has yet to be explored. Therefore, this study was conducted to investigate the effects of residence patterns relative to the workplace on the total fertility rate of working women.

**METHODS:**

Information on eligibility and healthcare utilization was obtained from the National Health Information Database between 2011 and 2015. The study participants were working women aged 15-49 years. We classified their residence relative to their workplace into 3 patterns: same municipality, same province, and different province. The total fertility rate was calculated and logistic regression was performed of childbirth according to residence pattern, adjusting for age, insurance contribution quartile, size of the workplace, year of birth, and province of residence.

**RESULTS:**

The total fertility rates of working women from 2011 to 2015 were 1.091, 1.139, 1.048, 1.073, and 1.103, respectively. The total fertility rate by residence pattern was highest in women residing in the same municipality as their workplace. After adjustment, the odds of childbirth in women from the same municipality and the same province were 21.6% and 16.0% higher than those of women residing in a different province, respectively.

**CONCLUSIONS:**

The total fertility rate was higher among women living near their workplace. Therefore, effective policy measures should be taken to promote the proximity of working women’s workplace and residence.

## INTRODUCTION

In 2017, the total fertility rate of women from the Republic of Korea (hereafter “Korea”) reached its lowest point, at approximately 1.05% [1], and the total fertility rate has remained below 1.30% from 2002 to 2017, which is referred to as “lowest-low fertility” [2]. Of the 11 countries in the Organization for Economic Cooperation and Development (OECD), only Korea has continued to demonstrate a lowest-low fertility status for the past 17 years [3]. Thus, the low fertility rate in Korea has gained much attention worldwide [4]. Despite the government’s efforts to encourage childbirth, the low birth rate in Korea has not improved. In fact, the number of births in the third quarter of 2018 has reached its lowest per quarter [5].

Eun [6] proposed 3 major causes of the low fertility rate in Korea: economic factors, improvements in women’s status, and strong traditional family values. The economic crisis, which began in late 1997, caused a serious problem in youth unemployment, which led to a decline in marriage and childbirth. The increase in women’s participation in economic activities may have affected the low fertility rate. Based on studies of women’s participation in economic activities in various countries, until the 1970s, women’s labour market participation had a negative correlation with birth rate. However, after that, the trend was weakened or reversed [7,8]. This difference may be due to the environment of the workplace, family, and country, but not women’s labour activity itself, which is important for fertility.

In preceding studies that attempted to identify factors affecting the fertility of working women, individual factors, such as occupational type and earning income, were considered as the main variables [9,10]. Some studies have addressed the effects of community-level factors, such as childcare infrastructure and unemployment rates [11,12]. Several policy options—such as financial incentives, child-related leave, childcare provision, and affordable and available housing—are known to increase fertility. Some countries provide social housing programs to support a childbearing environment. Despite the importance of housing policy, insufficient related research has been conducted [13]. In Korea, a study on the effects of work–family compatibility policies was conducted, reporting a limited understanding of the root cause of the low fertility rate [14]. In particular, very little research has investigated the environment related to the increasing employment rate of women in Korea.

As the number of working women has increased, the residence patterns of Koreans have also changed. Kwak [15] classified “weekend couples” into 4 types: those with the husband living apart on weekdays, those with the wife living apart on weekdays, those with the wife and child living apart on weekdays, and those living in multiple residences. Han [16] reported that women’s feeling of guilt relating to child care was higher in weekend couples where the separation was caused by women’s employment than in those where it was caused by men’s employment. Although various types of residence patterns exist and the psychological burden of parenting has increased, to our knowledge, no prior study has investigated associations between residence patterns and childbirth based on real-world data in Korea. Housing policy and residence patterns are closely related, and an important link also exists between childbearing support policies and residence patterns.

This study aimed to investigate the effects of residence patterns relative to the workplace on the total fertility rate of working women. We sought to test the hypothesis that fertility would be lower if a woman’s place of residence is far from her workplace. We used information from the National Health Information Database (NHID) of the National Health Insurance Service (NHIS), which is composed of objective administrative data on the whole population of Korea.

## MATERIALS AND METHODS

In this study, information on eligibility and healthcare utilization was obtained from the NHID between 2011 and 2015 (Data No. NHIS-2017-1-207). Korea has a national health insurance system, which covers the entire Korean population. The eligibility database includes socio-demographic variables, such as gender, age, region, and insurance contribution based on the income level of the insured, and the healthcare utilization database includes disease codes, procedure codes, and medication history [17]. Among the variables in the eligibility database, age, insurance contribution quartile, the size of an individual’s workplace (fewer than 5 employees, 5-29 employees, 30-299 employees, and 300 employees or more), and workplace and home addresses on January 1 of every year were analysed in this study. The insurance contribution quartile was calculated by dividing the amount of the insurance contribution of the study participants into equal quarters. In the healthcare utilization database, we used the procedure codes for birth, which can distinguish between births of singletons and twins. The study participants included employed insured women (working women) aged 15-49 years; that is, the ages for which the total fertility rate was calculated.

In this study, the total fertility rate was calculated annually, and a logistic regression analysis of childbirth was performed. First, total fertility rates were calculated using the sum of age-specific birth rates of women aged 15-49 years based on a mid-year population. We classified women’s residence into 3 patterns (same municipality, same province, and different province) according to differences in workplace and home addresses. In Korea’s system of administrative subdivisions, the province is the largest level, and each province has sub-municipalities. The total fertility rate was calculated according to each residence pattern, and a stratified analysis was performed according to the insurance contribution quartile. The total fertility rate of the whole Korean population was also calculated using the NHID to compare the fertility level of working and non-working women. Second, a logistic regression analysis of childbirth according to residence pattern was performed. The presence or absence of birth was considered as the dependent variable, and residence pattern was the independent variable. The adjusted variables included age, insurance contribution quartile, size of the workplace, year of the data (2011-2015), and province of residence (17 provinces). Age was the only continuous variable, and the remaining variables were considered as categorical variables. A p-value <0.05 was considered to indicate statistical significance, and SAS Enterprise Guide version 7.1 (SAS Institute Inc., Cary, NC, USA) was used.

### Ethics statement

All components and procedures of this study were approved by the Institutional Review Board (IRB) of the National Health Insurance Service (IRB No. Sa-2017-HR-05-001). This study used secondary data, and each case in the database was de-identified. Therefore, it was not possible to obtain informed consent from each patient.

## RESULTS

### General characteristics of the study participants

Table 1 shows the general characteristics of the study participants. The number of working women increased from 3,411,930 in 2011 to 4,074,680 in 2015. The number of those who experienced childbirth increased from 144,653 in 2011 to 152,696 in 2015. However, the birth rate decreased (from 4.2% to 3.8%). The proportions of women living in the same municipality, same province, and different province relative to their workplace in 2015 were 33.3%, 35.7%, and 31.0%, respectively. Furthermore, 13.6% of the workplaces had fewer than 5 employees, 31.1% had 5-29 employees, 26.2% had 30-299 employees, and 29.1% had 300 employees or more. The distribution of places of residence was as follows: 23.2% resided in Seoul metropolitan city, 25.0% in other metropolitan cities, and 51.8% in other areas (small cities and rural areas). The first to fourth quartiles of insurance contributions each contained 25.0% of the participants. Their average age increased from 34.2 years in 2011 to 35.1 years in 2015.

### Total fertility rate

**Figure 1.**
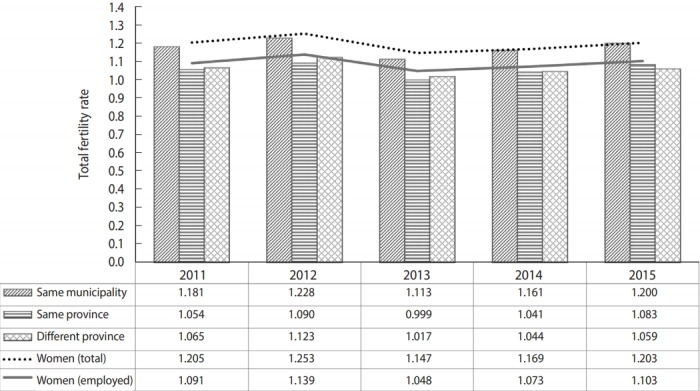
Total fertility rate of Korean women according to their residence pattern relative to their workplace (2011-2015).

The total fertility rates by year according to residence pattern are shown in Figure 1. From 2011 to 2015, the total fertility rates of women aged 15-49 years in Korea were 1.205, 1.253, 1.147, 1.169, and 1.203, respectively. The total fertility rates of working women from 2011 to 2015 were 1.091, 1.139, 1.048, 1.073, and 1.103, respectively, which were lower than those of the overall population of women in Korea. The total fertility rate in 2015 was highest (1.200) in women living in the same municipality as their workplace, followed by those living in the same province (1.083) and those living in a different province (1.059). Table 2 shows the total fertility rate by year according to the quartile of insurance contribution and residence type. In the case of women from the same municipality as their workplace, the total fertility rates of the first to fourth quartiles in 2015 were 0.969, 1.031, 1.285, and 1.586, respectively. This result indicates higher insurance contributions were positively associated with the total fertility rate. For women from the same province as their workplace, the total fertility rates of the first to fourth quartiles in 2015 were 0.858, 0.866, 1.075, and 1.355, respectively. For women residing in a different province from their workplace, the total fertility rates of the first to fourth quartiles in 2015 were 0.839, 0.809, 1.056, and 1.354, respectively. As of 2015, the total fertility rate of women from the same municipality as their workplace was the highest in all quartiles, followed by that of women from the same province and then from a different province.

### Logistic regression analysis of childbirth

Table 3 shows the results of the logistic regression analysis of childbirth. After adjusting for the relevant variables, adjusted odds ratio (aOR) for likelihood of childbirth of women from the same municipality as their workplace, and women from the same province was 1.22, and 1.16 (95% confidence interval [CI], 1.21 to 1.22, and 1.15 to 1.17) than women from a different province, respectively. According to the quartile of insurance contributions, aOR for the likelihood of childbirth of the second, third, and fourth quartiles was 1.15, 2.14, and 3.20 (95% CI, 1.14 to 1.16, 2.13 to 2.16, and 3.18 to 3.23) than in the first quartile, respectively. According to the size of the workplace, aOR of the companies with 5-29 employees, 30-299 employees, and 300 employees or more was 0.93, 0.78, and 0.77 (95% CI, 0.92 to 0.94, 0.78 to 0.79, and 0.76 to 0.77) than in those with fewer than 5 employees, respectively. The aOR of age by 1 year increase was 0.93 (95% CI, 0.93 to 0.93). A Poisson regression model was also analysed, but the logistic regression model showed higher fitness (Akaike information criterion of the logistic regression vs. the Poisson regression: 5,931,510.9 vs. 5,973,549.2).

## DISCUSSION

Based on this study, working women from the same municipality as their workplace had a higher birth rate than those from a different municipality or province. The differences according to the pattern of residence were consistent across all 4 quartiles of insurance contributions, and the total fertility rate of working women from the same municipality was close to that of the entire population of women in Korea. Even after the size of the workplace, insurance contribution quartile, and residential area were adjusted, residents living in the same municipality as their workplace had an approximately 21.6% higher likelihood of fertility than those living in a different province. Despite various environmental factors, such as childcare infrastructure, living near one’s workplace itself might increase the fertility rate.

This study is meaningful in that it analysed the impact of workplace location relative to residence in working women, which has not yet been approached in the Korean context in the low-birth-rate era. Methodologically, a descriptive statistical method was applied by comparing the total fertility rate, and the influencing factors were compared by logistic regression. As a result, various conclusions can be drawn. The hypothesis that the fertility rate of women living near the workplace would be high was verified through actual field data, and it was confirmed that the living environment is an important factor in the fertility trends of women.

The relative difference in the fertility rate according to the quartile of the insurance contribution, an indicator of income level, was the most significant, which supports the results of previous studies indicating that economic factors have caused the decrease in birth rate [6]. Based on a previous study of the distance between one’s work and residence, which was similar to this study, Zhang et al. [18] reported that the longer the commuting time of the husband, the lower the birth rate. Huinink & Feldhaus [19] reported a negative correlation between long-distance commuting and expectations of childbirth in women. Both studies had limitations because of a small sample size that included fewer than 10,000 people. Furthermore, Zhang et al. [18] did not address the growing number of women in the workplace, while Huinink & Feldhaus [19] analysed the intention to give birth, but not actual births. Therefore, this study can overcome the limitations of the previous studies.

According to a time-use survey conducted by the OECD, the commuting time of both men and women in Korea was the highest among 29 countries (26 OECD countries and China, India, and South Africa) [20]. For women, the average commuting time was about 42 minutes, which is about twice the OECD average of 22 minutes. The time spent by Korean women on caring for other members of the household was also comparable to that observed in 6 of 29 countries, suggesting that work–family balance is structurally challenging.

In various countries, strategies that encourage childbirth, such as subsidies, have proven effective [21,22]. In Korea, despite a temporary rebound in the fertility rate due to the implementation of childbirth support policies, the policy is still vulnerable, and its sustainability may be in question [23]. Another study also showed that Korean women’s working hours are long, which is a distinct factor in Korea compared to other countries [24]. In addition, women’s long working hours and commuting times may not have helped in properly implementing policies to encourage birth. Based on the results of this study, the proximity of women’s workplace and residence should be ensured as a way of effectively implementing such policies. Whereas the previous childbirth support policy was an ex-post-facto approach to support the costs of childcare after childbirth, a policy approach considering the location of women’s workplace relative to their residence would be a pre-activation policy. In order to increase the fertility rate in Korea, the Korean context—with a high rate of women’s economic activity—must be considered. The conclusion that working women’s residences should be located near their workplaces means that birth issues should be approached in terms of their relevance to housing policy.

There is a limitation in applying administrative differences, rather than commuting distances, due to the absence of available data. Even if the commuting distance is short, the administrative area may be different. On the contrary, a long commute may be possible even within the same the administrative area. Women’s place of residence was analysed at a specific time point in January, and the analysis presented herein did not reflect changes in the address of women’s workplace or residence after pregnancy. However, in order to perform the analysis properly from the methodological aspect of research design, it was necessary to set a specific time point. We focused on the differences between workplace and residence locations and assumed that other conditions related to childcare were similar. However, in a living environment that is independent from other regions, such as island regions (e.g., Jeju Island) or administrative capitals (e.g., Sejong), the relative likelihood of childbirth was higher even after adjusting for other factors. Thus, other factors may not have been sufficiently investigated in this study, which can be considered a limitation. Another limitation is that only the quantitative aspect of childbirth according to the location of women’s workplace and residence was analysed, while qualitative aspects of births, such as premature birth, were not covered. Women’s level of education was not considered because the NHID does not contain any directly relevant variables. There is a possibility of endogeneity in our model, but the intra-class correlation of residence pattern with fertility according to the region (at si/do levels), was only 0.03% in a hierarchical regression model. Therefore, a logistic regression model was used instead of a hierarchical model; however there may have been endogenous factors due to unobserved variables not included in this study. Further research should utilize information reflecting the actual distance between women’s workplace and residence and seek to further identify differences in impact relative to the time necessary to commute between those points.

In this study, we examined the effects of differences in distance between residence and workplace on the birth rate of working women, which has not been fully elucidated previously. Moreover, a notable aspect of this study is that information on health insurance claims from the NHID, which have mainly been used for clinical research, can be applied to social issues, such as childbirth. An appropriate approach for the situation in Korea—in which various policies have been made but not effectively implemented—was carried out in this study. The results will be used as a basis for future policies to address the low fertility rate.

## Figures and Tables

**Table 1.** General characteristics of the study participants

Characteristics	2011	2012	2013	2014	2015
Total	3,411,930 (100)	3,572,301 (100)	3,719,281 (100)	3,927,194 (100)	4,074,680 (100)
Childbirth	144,653 (4.2)	153,598 (4.3)	141,232 (3.8)	147,943 (3.8)	152,696 (3.8)
Residence pattern relative to workplace					
Same municipality	1,149,284 (33.7)	1,195,239 (33.5)	1,252,537 (33.7)	1,336,261 (34.0)	1,358,039 (33.3)
Same province	1,240,316 (36.4)	1,290,666 (36.1)	1,333,489 (35.9)	1,401,915 (35.7)	1,455,638 (35.7)
Different province	1,022,330 (30.0)	1,086,396 (30.4)	1,133,255 (30.5)	1,189,018 (30.3)	1,261,003 (31.0)
Employees in the company					
<5	427,659 (12.5)	441,196 (12.4)	480,212 (12.9)	521,830 (13.3)	554,333 (13.6)
5-29	1,077,525 (31.6)	1,132,227 (31.7)	1,176,699 (31.6)	1,215,261 (30.9)	1,266,861 (31.1)
30-299	970,962 (28.5)	1,001,325 (28.0)	1,027,235 (27.6)	1,059,368 (27.0)	1,066,931 (26.2)
≥300	935,784 (27.4)	997,553 (27.9)	1,035,135 (27.8)	1,130,735 (28.8)	1,186,555 (29.1)
Region					
Seoul metropolitan city	826,972 (24.2)	861,573 (24.1)	887,917 (23.9)	921,268 (23.5)	946,121 (23.2)
Other metropolitan city	857,144 (25.1)	895,978 (25.1)	937,379 (25.2)	978,920 (24.9)	1,019,840 (25.0)
Busan	229,864 (6.7)	237,544 (6.7)	242,852 (6.5)	251,532 (6.4)	261,960 (6.4)
Daegu	157,322 (4.6)	164,136 (4.6)	169,212 (4.6)	176,807 (4.5)	181,871 (4.5)
Incheon	194,693 (5.7)	206,428 (5.8)	217,378 (5.8)	228,329 (5.8)	237,903 (5.8)
Gwangju	99,969 (2.9)	104,803 (2.9)	109,481 (2.9)	113,659 (2.9)	118,261 (2.9)
Daejeon	106,754 (3.1)	111,169 (3.1)	115,819 (3.1)	121,491 (3.1)	125,631 (3.1)
Ulsan	68,542 (2.0)	71,898 (2.0)	75,121 (2.0)	78,100 (2.0)	81,065 (2.0)
Sejong	-	-	7,516 (0.2)	9,002 (0.2)	13,149 (0.3)
Other area	1,727,814 (50.7)	1,814,750 (50.8)	1,893,985 (50.9)	2,027,006 (51.6)	2,108,719 (51.8)
Gyeonggi-do	847,931 (24.9)	895,473 (25.1)	943,654 (25.4)	996,201 (25.4)	1,037,743 (25.5)
Gangwon-do	80,432 (2.4)	84,508 (2.4)	88,696 (2.4)	100,228 (2.6)	103,720 (2.6)
Chungcheongbuk-do	101,299 (3.0)	105,036 (2.9)	109,195 (2.9)	117,739 (3.0)	123,389 (3.0)
Chungcheongnam-do	124,764 (3.7)	131,255 (3.7)	131,946 (3.6)	145,704 (3.7)	149,674 (3.7)
Jeollabuk-do	103,375 (3.0)	107,653 (3.0)	112,052 (3.0)	120,928 (3.1)	125,166 (3.1)
Jeollanam-do	85,962 (2.5)	89,845 (2.5)	93,626 (2.5)	103,511 (2.6)	109,505 (2.7)
Gyeongsangbuk-do	148,662 (4.4)	155,602 (4.4)	160,154 (4.3)	169,192 (4.3)	174,370 (4.3)
Gyeongsangnam-do	201,006 (5.9)	209,121 (5.9)	215,944 (5.8)	229,475 (5.8)	238,563 (5.9)
Jeju-do	34,383 (1.0)	36,257 (1.0)	38,718 (1.0)	44,028 (1.1)	46,589 (1.1)
Insurance contribution (quartile)					
1st	850,123 (24.9)	914,087 (25.6)	929,444 (25.0)	986,423 (25.1)	1,018,829 (25.0)
2nd	847,367 (24.8)	871,205 (24.4)	930,554 (25.0)	977,265 (24.9)	1,018,683 (25.0)
3rd	861,498 (25.3)	893,952 (25.0)	929,417 (25.0)	981,693 (25.0)	1,018,531 (25.0)
4th	852,942 (25.0)	893,057 (25.0)	929,866 (25.0)	981,813 (25.0)	1,018,637 (25.0)
Age (mean±SD)	34.2±8.1	34.4±8.1	34.7±8.2	35.0±8.2	35.1±8.3

Values are presented as number (%). SD, standard deviation.

**Table 2.** Total fertility rates according to residence pattern relative to one’s workplace and insurance contribution (quartiles)

Year	n	Age, mean±SD	Total fertility rate			
			1st	2nd	3rd	4th
Same municipality						
2011	1,149,284	36.1±7.9	1.069	0.993	1.246	1.413
2012	1,195,239	36.3±7.9	1.110	1.039	1.299	1.468
2013	1,252,537	36.6 ±7.9	0.986	0.952	1.154	1.439
2014	1,336,261	36.9±8.0	0.992	0.977	1.240	1.442
2015	1,358,039	37.1±8.1	0.969	1.031	1.285	1.586
Same province						
2011	1,240,316	33.9±7.9	0.897	0.877	1.017	1.262
2012	1,290,666	34.1±7.9	0.944	0.869	1.060	1.318
2013	1,333,489	34.4±8.0	0.834	0.792	0.951	1.274
2014	1,401,915	34.7±8.1	0.844	0.831	1.026	1.309
2015	1,455,638	34.8±8.2	0.858	0.866	1.075	1.355
Different province						
2011	1,022,330	32.5±8.1	0.821	0.839	1.052	1.342
2012	1,086,396	32.7±8.2	0.906	0.866	1.101	1.427
2013	1,133,255	32.9±8.3	0.791	0.785	0.985	1.312
2014	1,189,018	33.3±8.3	0.799	0.809	1.065	1.292
2015	1,261,003	33.4±8.4	0.839	0.809	1.056	1.354

SD, standard deviation.

**Table 3.** Multivariate logistic regression for the likelihood of childbirth in working women (2011-2015)

Variables		aOR (95% CI)	SE of estimate	p-value
Residence patterns	Different province	1.00 (reference)		
	Same municipality	1.22 (1.21, 1.22)	0.002	<0.001
	Same province	1.16 (1.15, 1.17)	0.002	<0.001
Insurance contribution (quartile)	1st	1.00 (reference)		
	2nd	1.15 (1.14, 1.16)	0.002	<0.001
	3rd	2.14 (2.13, 2.16)	0.002	<0.001
	4th	3.20 (3.18, 3.23)	0.002	<0.001
Employees in the company	<5	1.00 (reference)		
	5-29	0.93 (0.92, 0.94)	0.002	<0.001
	30-299	0.78 (0.78, 0.79)	0.002	<0.001
	≥300	0.77 (0.76, 0.77)	0.002	<0.001
Age		0.93 (0.93, 0.93)	<0.001	<0.001
Year	2011	1.00 (reference)		
	2012	1.03 (1.02, 1.04)	0.002	<0.001
	2013	0.92 (0.91, 0.93)	0.002	<0.001
	2014	0.94 (0.93, 0.94)	0.002	<0.001
	2015	0.94 (0.93, 0.95)	0.002	<0.001
Region	Seoul	1.00 (reference)		
	Busan	1.14 (1.13, 1.15)	0.005	<0.001
	Daegu	1.20 (1.19, 1.22)	0.006	0.291
	Incheon	1.09 (1.08, 1.10)	0.005	<0.001
	Gwangju	1.39 (1.37, 1.41)	0.007	<0.001
	Daejeon	1.29 (1.27, 1.31)	0.007	<0.001
	Ulsan	1.21 (1.19, 1.24)	0.008	0.602
	Sejong	1.41 (1.33, 1.49)	0.027	<0.001
	Gyeonggi-do	1.16 (1.15, 1.17)	0.003	<0.001
	Gangwon-do	1.12 (1.11, 1.14)	0.008	<0.001
	Chungcheongbuk-do	1.15 (1.13, 1.17)	0.007	<0.001
	Chungcheongnam-do	1.08 (1.07, 1.10)	0.006	<0.001
	Jeollabuk-do	1.24 (1.22, 1.26)	0.007	<0.001
	Jeollanam-do	1.31 (1.29, 1.33)	0.007	<0.001
	Gyeongsangbuk-do	1.09 (1.08, 1.11)	0.006	<0.001
	Gyeongsangnam-do	1.26 (1.24, 1.27)	0.005	<0.001
	Jeju-do	1.52 (1.48, 1.55)	0.010	<0.001
AIC	5,931,510.90			

aOR, adjusted odds ratio; CI, confidence interval; SE, standard error; AIC, Akaike information criterion.
